# Preferential Nucleation during Polymorphic Transformations

**DOI:** 10.1038/srep30860

**Published:** 2016-08-03

**Authors:** H. Sharma, J. Sietsma, S. E. Offerman

**Affiliations:** 1 Department of Materials Science and Engineering, Delft University of Technology, Mekelweg 2, 2628 CD Delft, The Netherlands

## Abstract

Polymorphism is the ability of a solid material to exist in more than one phase or crystal structure. Polymorphism may occur in metals, alloys, ceramics, minerals, polymers, and pharmaceutical substances. Unresolved are the conditions for preferential nucleation during polymorphic transformations in which structural relationships or special crystallographic orientation relationships (OR’s) form between the nucleus and surrounding matrix grains. We measured *in-situ* and simultaneously the nucleation rates of grains that have zero, one, two, three and four special OR’s with the surrounding parent grains. These experiments show a trend in which the activation energy for nucleation becomes smaller – and therefore nucleation more probable - with increasing number of special OR’s. These insights contribute to steering the processing of polymorphic materials with tailored properties, since preferential nucleation affects which crystal structure forms, the average grain size and texture of the material, and thereby - to a large extent - the final properties of the material.

Polymorphism is observed in a wide variety of material classes^1^,^2^,^3^,^4^,^5^,^6^. The prevailing crystal structure of a solid depends on the temperature, external pressure, and the kinetics of the transformation^1^,^2^. The nucleation kinetics of polymorphic transformations affect which (meta-stable) crystal structure forms^7^,^8^, the average grain size after the transformation^9^,^10^, the texture^11^, and thereby - to a large extent - the final properties of the material. Certain conditions are known to stimulate nucleation through mechanisms that reduce the activation energy. Two important conditions for preferential solid-state nucleation are: i) the presence of heterogeneous sites in the material^12^,^13^,^14^ and ii) the existence of structural relationships or special crystallographic orientation relationships (OR’s) between the two crystal structures^11^,^15^,^16^,^17^.

For the first case, the underlying mechanism is the elimination of grain boundary area of the matrix during the nucleation, which releases energy of the system and effectively lowers the activation energy for nucleation^12^. Therefore, the nucleation preferentially takes place at junctions where 4 grains meet (corners) followed by junctions where 3 (edges), and 2 (faces) grains meet. For the second case, the situation is more complex, because of two competing mechanisms: the minimization of interphase boundary energy and the minimization of strain energy. On the one hand, the formation of interphase boundaries with special OR’s may result in (semi-)coherent interphase boundaries with lower energy than incoherent interphase boundaries^18^, which in turn lowers the activation energy for nucleation. On the other hand, the formation of coherent interfaces results in a higher strain energy^19^, which increases the activation energy for nucleation compared to nuclei with incoherent interfaces. The relationship between the activation energy and the number of special OR’s has not been derived from the nucleation theory without a priori assumptions. The complexity of the problem is threefold: (a) in practice, solid-state nucleation takes place at heterogeneous sites^12^, (b) the interfacial energy between a nucleus and the matrix is generally anisotropic, and (c) the coherency strain is generally anisotropic^20^. Here, we present an experimental study into the relationship between the activation energy (indicative for preferred nucleation) and the number of special OR’s.

An experimental challenge is to measure the crystallographic orientations, positions, and volumes of new grains and their surrounding parent grains during a polymorphic transformation. Here, we present *in-situ* three-dimensional x-ray diffraction (3DXRD) microscopy measurements (see supplementary Fig. S1) of the ferrite (α) to austenite (γ) transformation in steel during heating, which we performed at the European Synchrotron Radiation Facility^21^,^22^. The steel specimen (see supplementary Fig. S2) is given a step-wise heat-treatment (see supplementary Fig. S3) in a furnace that we specifically designed for measurements with synchrotron x-rays^23^.

## Results and Discussion

### Evolution microstructure and distribution of crystallographic orientation relationships

**Figure 1:**
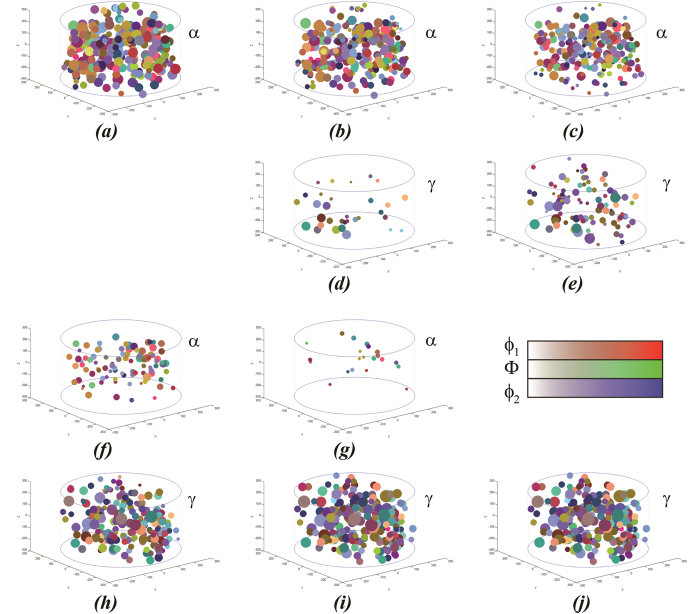
Evolution of the microstructure at the level of grains as measured during the α → γ phase transformation. Microstructure (**a**) before the phase transformation at 1103 K, (**b**,**d**) 1109 K (**c**,**e**), 1113 K, (**f**,**h**) 1117 K, (**g**,**i**) 1119 K, and (**j**) after the phase transformation at 1121 K. Position of (**a**–**c**,**f**,**g**) the α grains and (**d**,**e**,**h**–**j**) the γ grains. The axis units are micrometers. Grains are represented as spheres. The size of each marker is equal to the size of the corresponding grain divided by 2.5, which is needed to visualize the microstructure. The two blue circles in each plot indicate the size of the sample cylinder (500 μm diameter). Colours represent orientation according to the colour legend (Euler angles).

Figure 1 shows the evolution of the microstructure at the level of grains as measured during the ferrite to austenite phase transformation in three dimensions (3D). The raw 3DXRD-data is converted into the volume, position in the specimen (center of mass), and crystallographic orientation of each ferrite and austenite grain in the illuminated sample volume. The method for this conversion is described elsewhere^24^,^25^. During the transformation the temperature interval at which the austenite grains are first detected is also recorded. From this information we derive for each austenite grain: (a) the surrounding ferrite grains, (b) the crystallographic orientation relationships between the austenite grain and its ferrite neighbors, and (c) the nucleation temperature.

**Figure 2:**
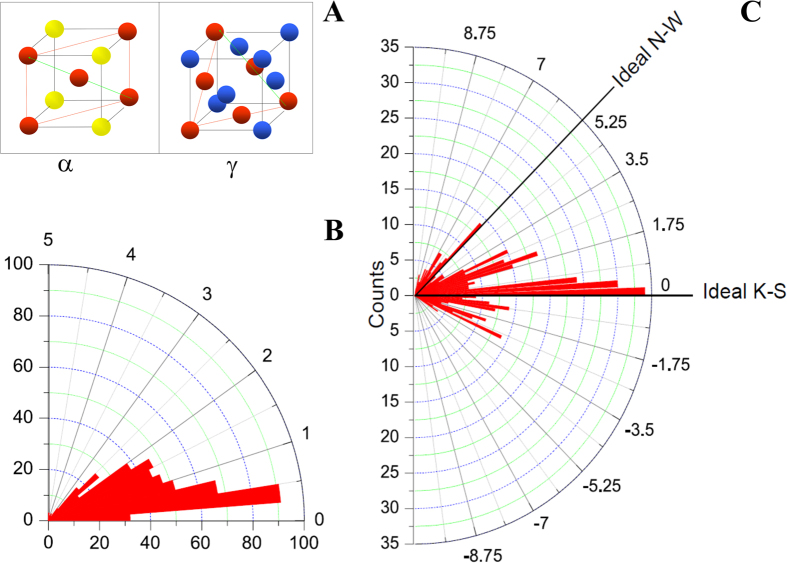
Crystallographic orientation relationships (OR’s) between ferrite (α) and austenite (γ). (**A**) Schematic illustration of the special Kurdjumov-Sachs (K-S) OR. The spheres indicate the positions of the atoms. The parallel plane condition {110}_α_//{111}_γ_ is illustrated with the red planes and the parallel direction condition 〈111〉_α_//〈110〉_γ_ is illustrated with the green arrows. Polar histogram of the number of the observed OR’s as a function of the deviation angle from the parallel (**B**) plane and (**C**) direction conditions. The horizontal axis (0°) in (**B**) represents the parallel plane condition {110}_α_//{111}_γ_ for both the K-S and Nishiyama-Wasserman (N-W) OR’s. The horizontal axis (0°) in (**C**) represents the parallel direction condition for the ideal K-S OR: 〈111〉_α_//〈110〉_γ_.

Figure 2(A) schematically shows a special crystallographic orientation relation between the body-centered cubic (BCC) ferrite phase and the face-centered cubic (FCC) austenite phase, which is known as the Kurdjumov-Sachs (K-S) OR. In this study, we define an OR as special in case the minimum angle between the {110}_α_- and the {111}_γ_-planes of the ferrite and austenite grains, respectively, is less than 3° and the corresponding angle between the 〈111〉_α_- and 〈110〉_γ_-directions is less than 12°. These angular ranges cover the two ideal special crystallographic OR’s in steel: K-S and Nishiyama-Wasserman (N-W). Furthermore, Fig. 2 shows in two polar histograms the number of observed OR’s between ferrite and austenite grain pairs as a function of the deviation angles from (a) the parallel plane condition and (b) the parallel direction condition. A distribution of special OR’s is observed around the ideal K-S and N-W OR’s in Fig. 2.

About 93% of the nuclei have one or more special OR’s with the surrounding matrix grains, which is in line with earlier findings^15^,^16^,^17^. Further breakdown of the data shows that the number of austenite grains that have zero, one, two, three, and four special OR’s with the matrix ferrite grains is 18, 104, 68, 40, and 38, respectively. Noteworthy is that the frequency of occurrence of nuclei having a special OR with three and four neighbors is 29%, which is high compared to earlier findings in an Fe-Co alloy^17^. This high frequency cannot be explained by nuclei with 3 or 4 special orientation relationships having a higher number of neighbours than nuclei with 0, 1 or 2 special OR’s, which follows directly from the observed numbers of neighbouring grains for the different types of nuclei. Instead, the high frequency indicates the occurrence of a texture memory effect in this steel^11^.

### Effect of the number of special OR’s on the nucleation rates

**Figure 3:**
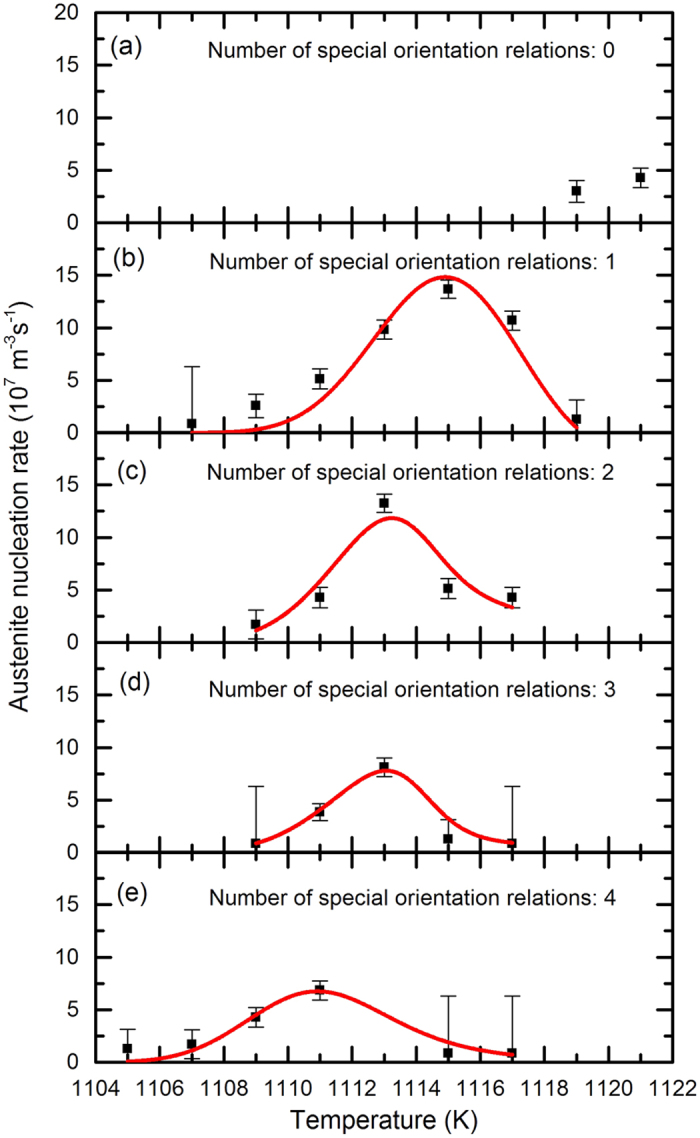
The austenite nucleation rate as a function of temperature. The number of special OR’s is (**a**) 0, (**b**) 1, (**c**) 2, (**d**) 3, and (**e**) 4. The solid lines indicate fits of the classical nucleation theory to the experimental data (squares).

Figure 3 shows the measured austenite nucleation rates as a function of temperature for five cases: austenite grains that have special OR’s with either zero, one, two, three, or four surrounding ferrite grains. The classical nucleation theory is fitted to the measured nucleation rates. The classical nucleation theory describes the total nucleation rate per unit volume as the sum of the nucleation rates at different potential nucleation sites as given by^12^


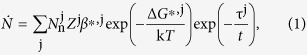


where the index *j* refers to the type of potential nucleation site. Since polycrystalline materials can be composed of many different grain corners, edges, and faces with different shapes and grain boundary energies, polycrystalline materials in principle contain a large variety of potential nucleation sites. The Zeldovich factor represents the influence of subcritical nuclei growing by thermal activation to the critical dimension. It is given by 
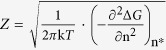
, where Δ*G* is the change in Gibbs free energy upon the formation of a (sub-critical) nucleus with *n* atoms and n* is the number of atoms in the critical nucleus. The other symbols are described in the manuscript. Δ*G* depends on the shape of the critical nucleus. The Zeldovich factor *Z* is assumed to be constant, because the rate of formation of critical nuclei and their growth to a supercritical size are similar. The frequency factor β^*^ is proportional to (*kT*/*h*)exp[−*Q*_D_/(*kT*)], where *k* is the Boltzmann constant, *h* the Planck constant, and *T* the temperature^5^. The mobility of the iron atoms in the paramagnetic ferrite phase is taken into account by the activation energy for self-diffusion^26^ *Q*_D_ = 3.93×10^−19^ J.

The density *N*_n_ of potential nucleation sites can decrease during the ferrite-to-austenite transformation due to: 1) the nucleation process itself and 2) the consumption of potential nucleation sites by growing grains. For the first case, we assume that the actual density of potential nucleation sites changes with temperature in the same way as the measured density of potential nucleation sites 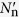 (see supplementary Fig. S4), except for a constant scaling factor *S*, i.e. 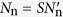. For the second case, the decrease of number of potential nucleation sites is proportional to (1 − *f*^ γ^), with *f*^γ^ the austenite fraction, which we measured (see supplementary Fig. S5) simultaneously during the transformation. The last exponential factor in equation (1) expresses the time-dependent part of the nucleation rate, where τ is the incubation time for nucleation, and *t* the time. For the given experimental conditions this exponential term is very close to one. The activation energy for nucleation ΔG^*^ is given by^9^,^27^


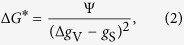


which implicitly defines the Ψ-parameter that contains information about the shape of the critical nucleus and interphase energies between the nucleus and the matrix grains and the energy that is released to the system due to the removal of grain boundary area between matrix grains due to the formation of a nucleus. The shape of a critical nucleus during a solid-state phase transformation depends on: 1) the characteristics of the (heterogeneous) potential nucleation site^12^, 2) the anisotropic interface energy between the matrix and the nucleus^12^, and 3) the anisotropic elastic/strain energy^20^. The mechanism behind the effect of the strain energy is that the density of a new phase and the corresponding parent phase is in general different, so elastic energy is generated during nucleation to accommodate the lattice mismatch between a nucleus and the matrix.

The driving force for nucleation, Δ*g*_V_, is the difference in Gibbs free energy per unit volume between the ferrite and the austenite, which is calculated with Thermo-Calc^®^ under para-equilibrium conditions (see supplementary Fig. S6)^28^. The misfit strain energy, *g*_S_, includes the dilatational strain energy (due to the difference in the average atomic volume in the nucleus and the matrix) and shear strain energy (due to the difference in the structure of the two crystals). The misfit strain energy *g*_S_ effectively reduces Δ*g*_V_ and thus increases the transformation start temperature with respect to the para-equilibrium condition (1024 K in this case). The misfit strain energy for the nucleation is estimated to be 10^7^ J/m^3^ from earlier work^19^, which corresponds very well to the driving force for nucleation at the temperature at which the transformation starts experimentally (see supplementary Fig. S6), *T*_α−γ_ = 1105 K: Δ*g*_V_ = 1.1 · 10^7^ J/m^3^. Therefore, at a temperature of 1105 K, the condition for the start of the transformation is fulfilled, i.e. Δ*g*_V_ > *g*_S_.

The temperature dependent factors of the nucleation rate of one type of nucleus can thus be written as


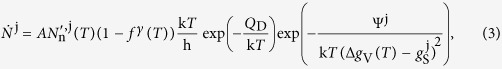


where *A* = (*S*/*C*)*Z*exp(−τ/*t*). The constant *C* is related to the time scale of nucleation and is introduced due to the measurement method. Equation (3) is used for fitting the measured nucleation rates with *A* and Ψ as fitting parameters. 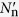
(*T*) and *f*^γ^(*T*) are taken from the measurements, Δ*g*_V_ (T) is calculated with the thermodynamic software package Thermo-Calc (see supplementary Fig. S6), and *Q*_D_ and *g*_S_ have the previously mentioned values^19^,^26^. The solid curves in Fig. 3 show the fits of the measured nucleation rate to equation (3). The classical nucleation theory describes the observed nucleation rates very well.

### Nucleation becomes more probable with increasing number of special OR’s

**Figure 4:**
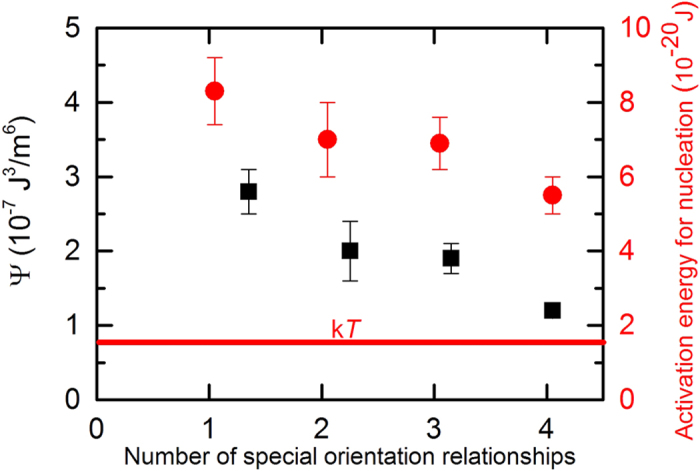
Critical nucleus parameters. The fitted values for the Ψ-parameter (squares) and the activation energy for nucleation (circles) as a function of the number of special OR’s between austenite and ferrite grains. Note: the error bar of the value for the Ψ-parameter for four special OR’s is equal to the symbol size.

Figure 4 shows the values of Ψ as a function of the number of special OR’s for the different types of nuclei. A trend can be observed in Fig. 4, in which Ψ decreases with increasing number of special OR’s. The absolute value for Ψ that we obtain from the fits is of the order of 10^−7^ J^3^/m^6^, which lies in the range of values that were reported earlier^9^,^17^: 10^−4^–10^−8^ J^3^/m^6^. The absolute values for the fitted Ψ-parameter are very sensitive to the value for *g*_S_, which we take from the literature^19^ and which is only reported for specific nucleus geometries. Changing *g*_S_ from 0 to 1.1 · 10^7^ J/m^3^ (the driving force at the start of the transformation), changes the value for Ψ from 10^−4^ to 10^−8^ J^3^/m^6^. However, the trend of Ψ decreasing with increasing number of special OR’s stays the same in case the strain energy *g*_S_ is (approximately) the same for all nuclei with 1–4 special OR’s. In this study, the strain energy *g*_S_ can be assumed to be the same for all nuclei with 1–4 special OR’s, since the nucleation start temperature is the same within 4 K, see Fig. 3.

Figure 4 also shows the activation energy ΔG^*^ for nucleation as a function of the number of special OR’s for the different types of nuclei. The values for ΔG^*^ are derived from the fit-parameter Ψ according to Eqn. (2), where the driving force for nucleation is taken at the temperature at which the nucleation rate is maximum. The activation energy for nucleation varies between 3 k*T* and 6 k*T*. Figure 4 shows a trend in which the activation energy ΔG^*^ for nucleation becomes smaller – and therefore nucleation more probable - with increasing number of special OR’s.

## Summarizing statement

The probability for nucleation during polymorphic transformations increases with the number of special crystallographic orientation relationships between nucleus and surrounding matrix grains.

## Methods

### Material

The steel was obtained by using vacuum induction casting in order to get a high-purity steel with a homogeneous composition (in wt.%) of 0.011 carbon (C), 0.87 manganese (Mn), 0.083 titanium (Ti) and the rest iron (Fe). The concentration of impurities is lower than 200 ppm for all other elements combined. The composition of the sample was determined using both electron probe micro-analysis (EPMA) and wet chemical analysis. No heterogeneities were detected.

### 3DXRD-measurements

The 3DXRD experiment was carried out at beamline ID11 of the European Synchrotron Radiation Facility (ESRF), Grenoble, France. Supplementary Fig. S1 shows schematically the experimental setup and defines the angles θ, η, and ω. The geometry of the steel sample is manufactured by using electro-discharge machining (EDM) with the dimensions shown in supplementary Fig. S2 in order to perfectly fit in the furnace described in ref. 23. In this way, the sample could freely expand/contract during the heat-treatment without introducing thermal strains into the material. The sample had a change in diameter from 1 mm to 1.5 mm in the middle, which was used to as a reference to define the exact position of the x-ray beam w.r.t. the specimen (by scanning with the X-ray beam). An S-type thermocouple was spot-welded to the top of the sample for accurate temperature control. The sample chamber was purged with helium and sealed at a pressure of 0.4 atm. The X-ray beam, 500 μm high and 500 μm wide with energy equal to 88.005 keV, calibrated using a Pb-foil, was incident on the sample at the location depicted in supplementary Fig. S2. Since nucleation and growth at the surface are essentially different from in the bulk, the beam size chosen was smaller than the diameter of the sample, see supplementary Fig. S2. Only that part of the volume of the sample is investigated and analyzed that was continuously illuminated by the beam as we rotated the specimen (henceforth called the ‘cylinder’). We make a distinction between the diffraction signal from the ‘cylinder’ volume and parts of the sample volume that were only instantaneously illuminated by the beam during rotation of the sample (see 5 sections below).

During the experiment, so-called ‘Friedel 3D measurements’ were carried out at each step. In essence, the Friedel 3D-measurements determine the crystallographic orientations, positions, and volumes of new grains and their surrounding parent grains. We do this according to a method that we developed^24^,^25^, which makes use of Friedel-pairs. Friedel-pairs are reflections from the (*hkl*)- and the 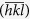-planes. We use the Friedel pairs to correct for imperfections in the alignment of the set-up: 1) possible tilt of the axis of rotation of the sample and 2) possible detector tilt with respect to the direction of the incoming x-ray beam (which ideally is perpendicular to both)^24^,^25^.

Each Friedel 3D measurement consisted of rotating the 3DXRD furnace over a total angle of 170°, 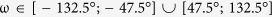. Diffraction images were recorded using a FReLoN detector during every 0.3° rotation with an exposure time of 0.2 s. The pixel size of the FReLoN detector is 50 μm, which is around 10 times smaller than the radius of the sample. We have taken into account that the point-spread-function of the FReLoN detector smears out the intensity of a diffraction spot over multiple pixels (typically 50–250 pixels). We use a three-dimensional Gaussian function for fitting the peak shape of the individual diffraction spots. This means that the positions of the centres of mass of the diffraction spots (expressed in the θ-, η-, and ω-angles, see supplementary Fig. 1 for definitions) can be determined with sub-pixel accuracy. We have performed simulations to estimate the error in the position of the grains for typical experimental conditions^24^,^25^, resulting in a value of 2–3 micrometre.

A Friedel 3D measurement required a total of approx. 13 min to complete. The sample to detector distance was calibrated in such a way that four complete diffraction rings of the austenite phase and three complete diffraction rings of the ferrite phase were observed in the diffraction images. For these experimental settings, 29–37 or 36–45 diffraction spots would be observed from each individual ferrite or austenite grain, respectively, depending on the orientation of the grain.

### Heat treatment

The pre-heat treatment of the sample involved slow heating to 1273 K, austenitization at 1273 K for 45 min in order to obtain coarse γ grain structure and cooling to room temperature. During the pre-heat treatment, the α → γ transformation range was found to lie between approximate 1103 K and 1125 K. After an initial Friedel 3D measurement at room temperature (RT), the sample is heat-treated in the following way, see supplementary Fig. S3. The sample is first heated in 5 min to a temperature 2 K below the transformation start temperature, followed by isothermal holding for 23 min. After 10 min isothermal holding to wait for the microstructure to stabilize, a Friedel 3D measurement was carried out in 13 minutes to measure the position, volume and crystallographic orientation of each ferrite grain. Thereafter, the temperature of the sample was increased by 2 K in 2 s. At the raised temperature, another Friedel 3D measurement was carried out after 10 min. in order to capture the microstructure of the sample after stabilization: the position, volume and crystallographic orientation of each ferrite and austenite grain. We have monitored the evolution of the transformation during the holding time of 10 minutes by the diffraction measurements, observing that the transformation came to a completion within 10 minutes of annealing between the temperature steps. This procedure, heating by 2 K, waiting for 10 min followed by a Friedel 3D measurement, was repeated up to a temperature of 1125 K was reached. The sample was then heated to a temperature of 1173 K in 60 s and held isothermally for 130 min, during which 10 repeated Friedel 3D measurements were carried out, followed by cooling to room temperature. The heat treatment is shown schematically in supplementary Fig. S3.

### 3DXRD Data-analysis

In total, 23 Friedel 3D measurements are carried out, meaning that information about the microstructure of the sample is obtained at 23 points during the heat-treatment. From each Friedel 3D measurement the volume, the centre-of-mass position and the orientation of each grain in the illuminated volume are determined. For the 3DXRD-data-analysis we followed the procedure that we presented in refs 24,25 in order to characterize the diffraction spots from the raw diffraction images. A more detailed description is given in the supplementary information about the analysis of the 3DXRD-data for this specific type of experiment.

## Additional Information

**How to cite this article**: Sharma, H. *et al*. Preferential Nucleation during Polymorphic Transformations. *Sci. Rep*. **6**, 30860; doi: 10.1038/srep30860 (2016).

## Supplementary Material

Supplementary Information
