# Supplementary material for: Preferential Nucleation during Polymorphic Transformations
Source: Sci Rep. 2016 Aug 3;6:30860. doi: 10.1038/srep30860 (PMC4971480; doi:10.1038/srep30860)
Supplement: Supplementary Information [file srep30860-s1.pdf]

Supplementary information

Preferential Nucleation during Polymorphic Transformations

H. Sharma[1,†], J. Sietsma[1], and S. E. Offerman[1,*]

[1] Department of Materials Science and Engineering, Delft University of Technology, Mekelweg 2, 2628 CD Delft, The Netherlands

[†] Present Address: Advanced Photon Source, Argonne National Laboratory, 9700 South Cass Ave., Argonne, IL 60439, USA

* Corresponding author: S.E.Offerman@tudelft.nl

This section contains the following supplementary information:

1. Supplementary Figs. S1-8, and Table S1
2. Intermediate results and discussion
3. Additional results and discussion
4. Details of the 3DXRD data-analysis.

Supplementary Figs. S1-8

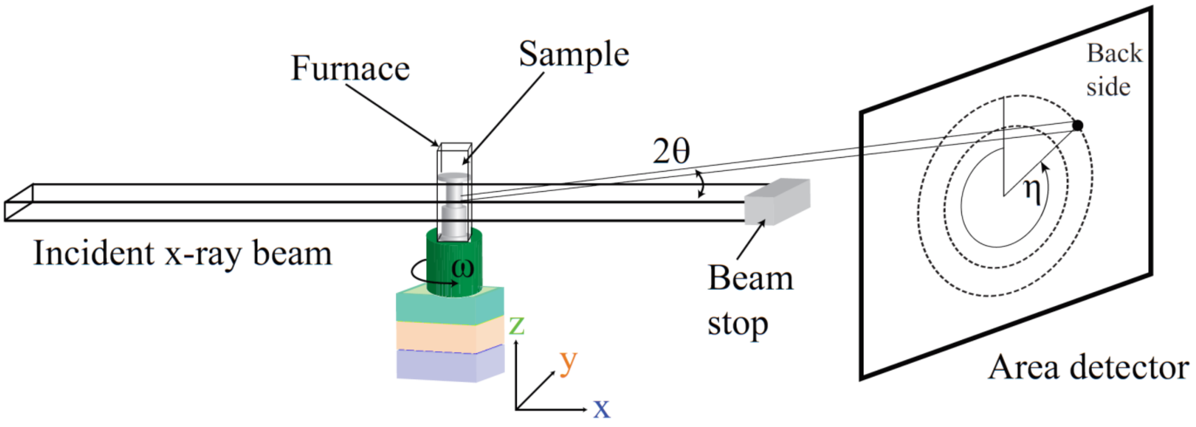

Fig. S1. Schematic showing the experimental setup of the 3DXRD microscopy. The dimensions are not to scale. The angles 2θ, ω and η are defined.

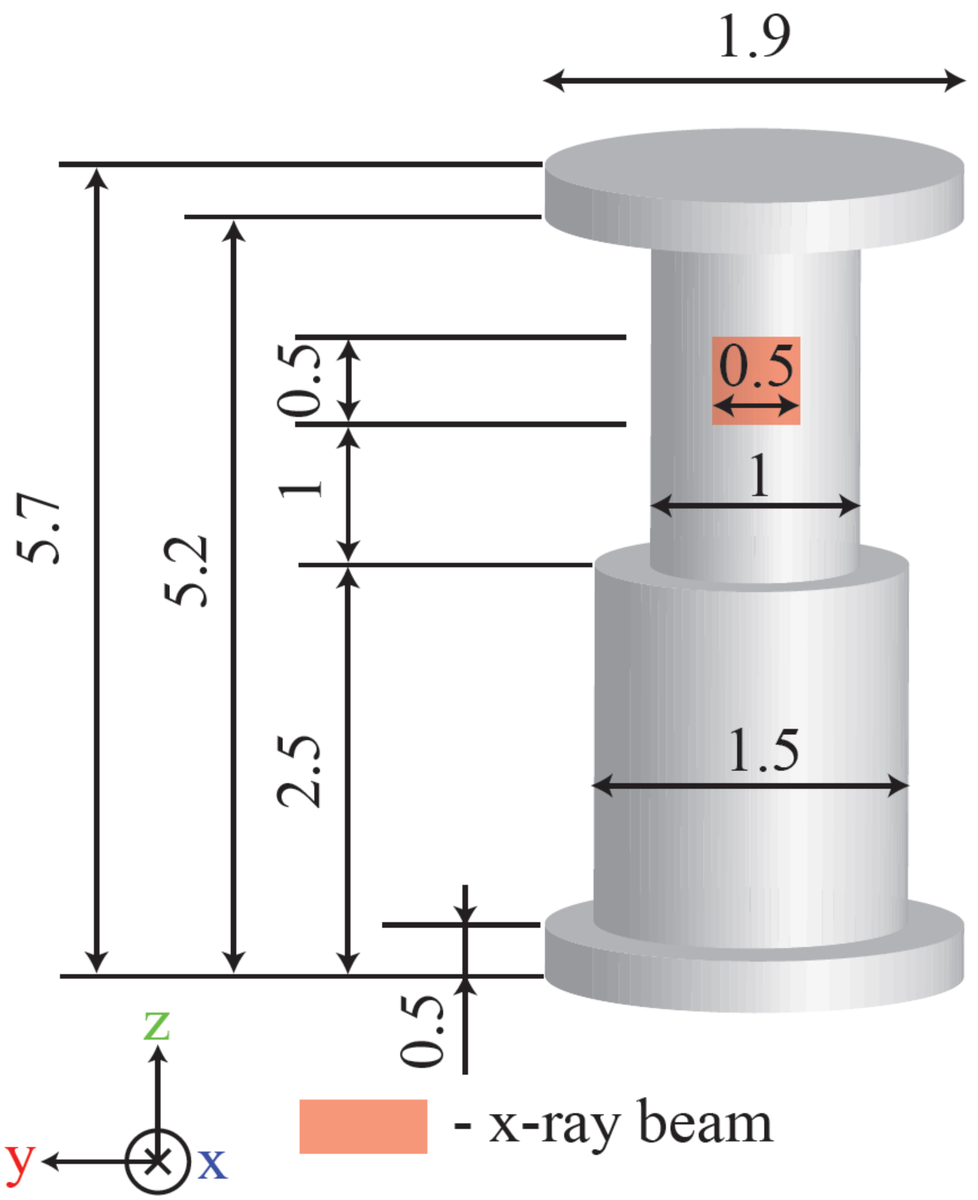

Figure S2: Schematic geometry of the sample. The location of the X-ray beam is highlighted. The dimensions are in mm.

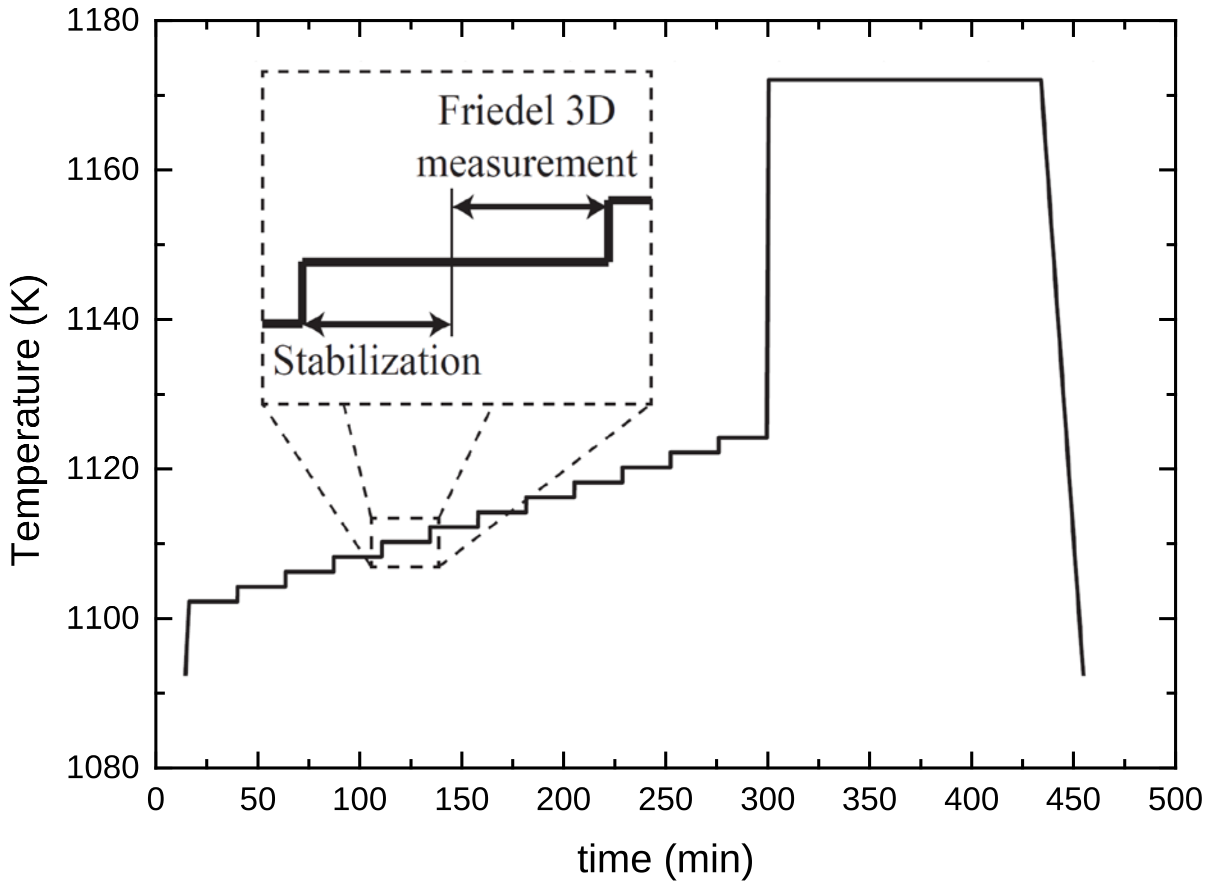

Figure S3. Schematic of the heat-treatment carried out on the sample. During the interrupted heating from 830 °C to 852 °C in 2 °C steps, after a stabilization step for 10 min, a Friedel 3D measurement is carried out at each step. At the isothermal annealing temperature of 900 °C, Friedel 3D measurements were carried out a total of 10 times.

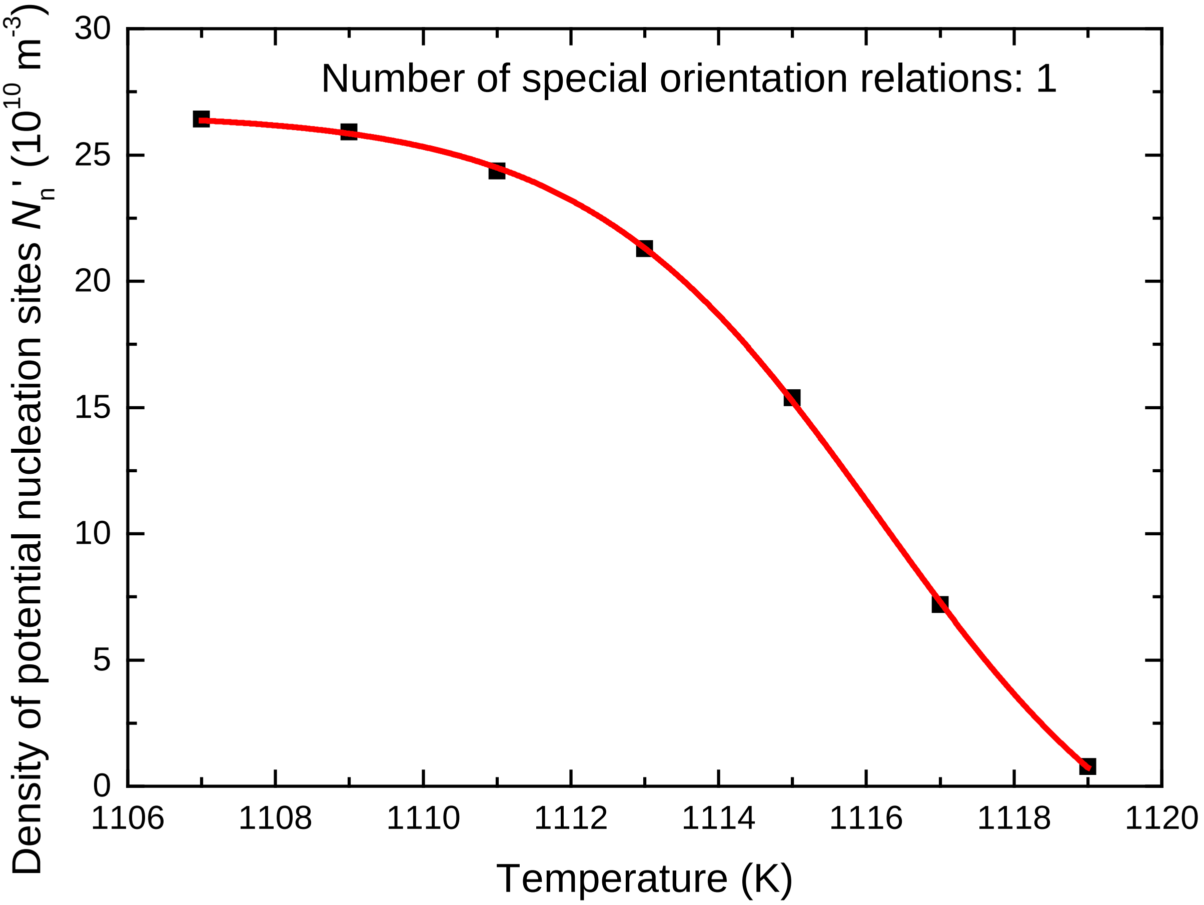

Figure S4: Density of potential nucleation sites $N_n^{'}$ as a function of temperature for OR1 as derived from the measurements.

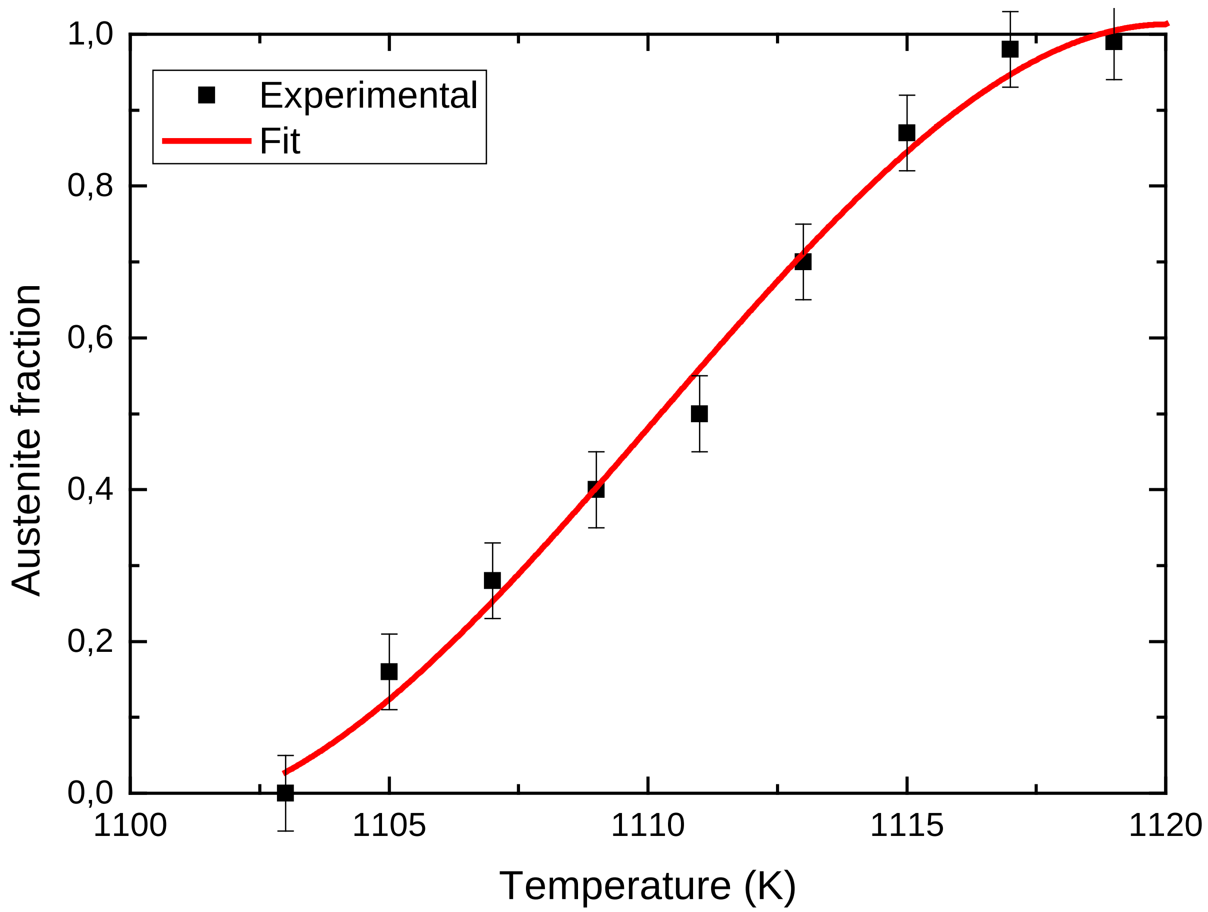

Figure S5: Measured austenite (γ) fraction as a function of temperature.

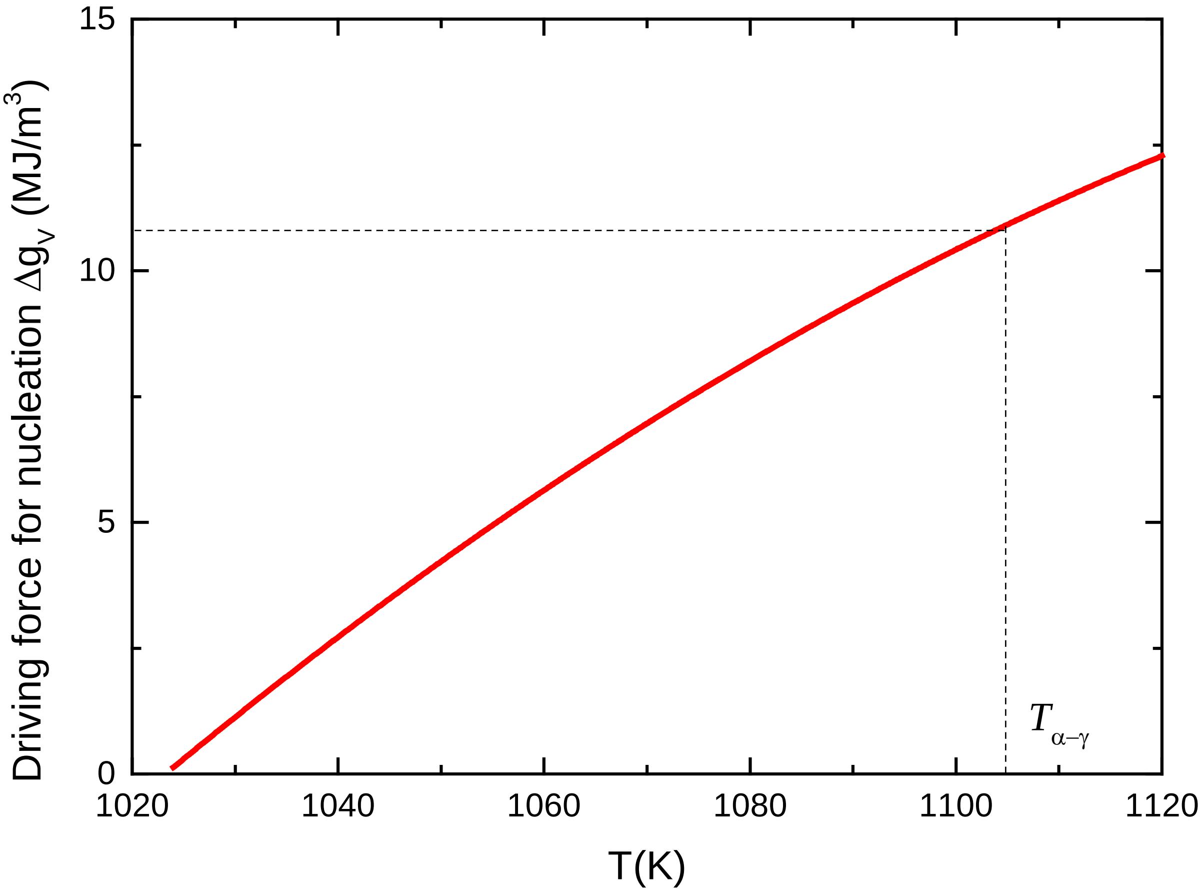

Figure S6: Driving force for nucleation as a function of temperature. The driving force for nucleation at the temperature $T_{\alpha\text{-}\gamma}$ at which the ferrite-to-austenite transformation starts is indicated by the dashed lines.

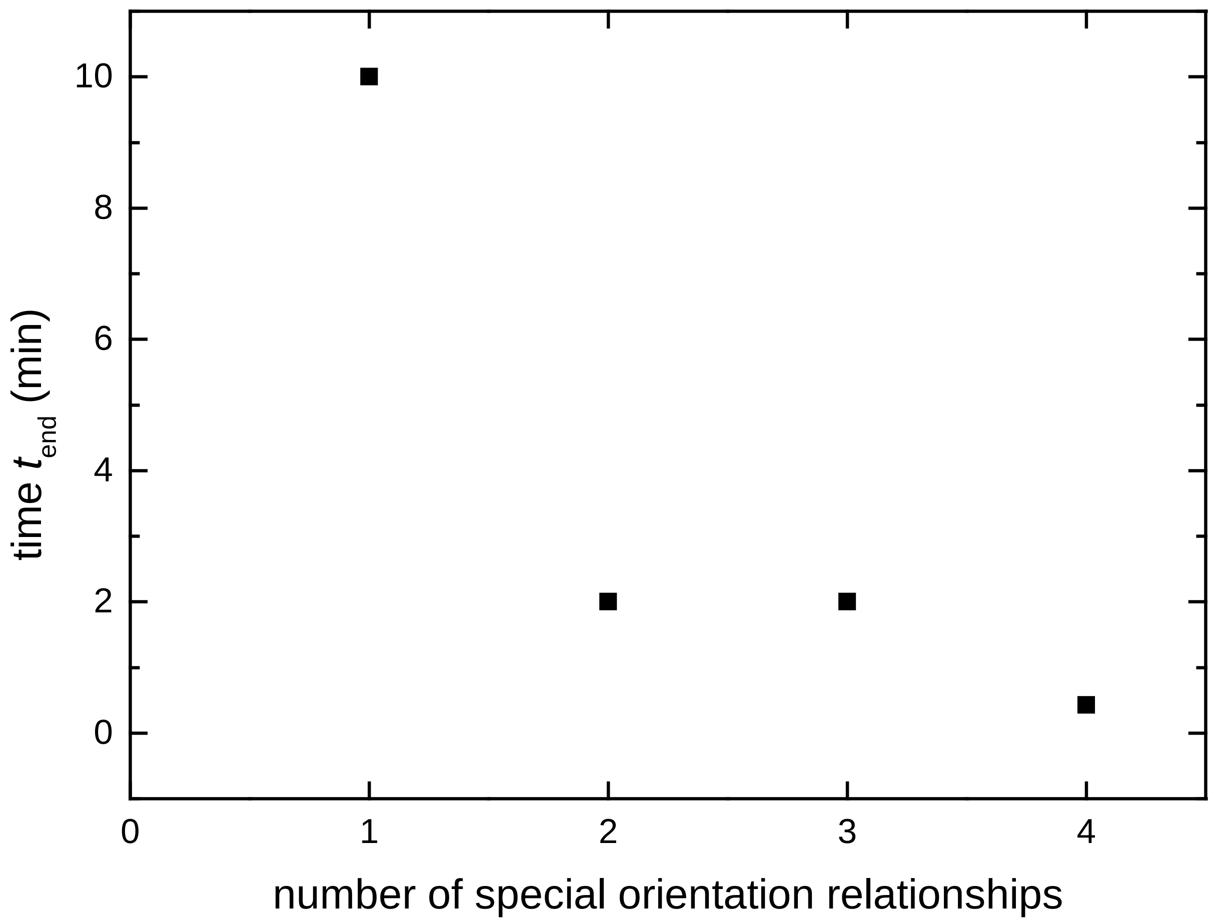

Figure S7: The maximum time t_{end} at which the nucleation process ends during isothermal holding as a function of the number of special OR's. This time t_{end} is derived from the fit-parameter A.

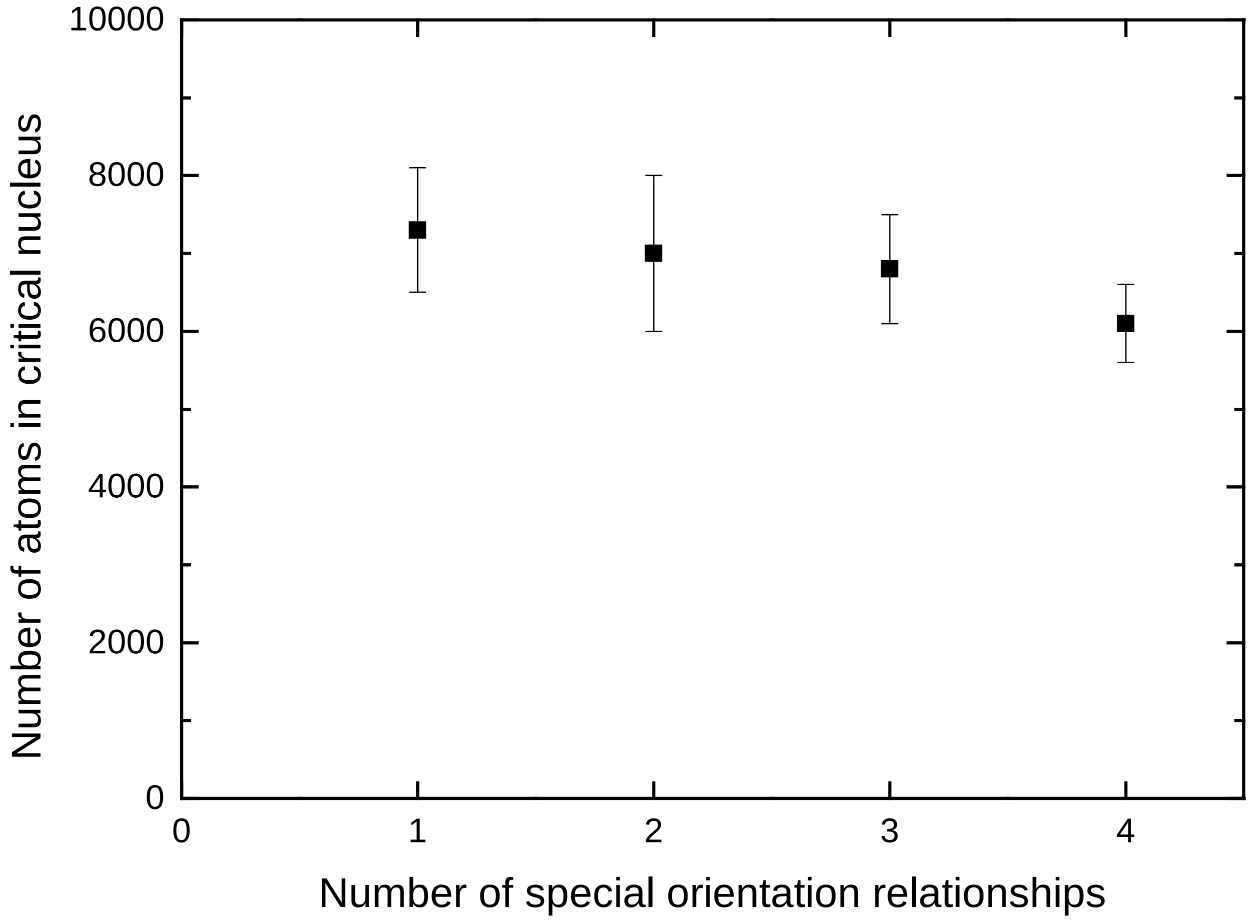

Fig. S8. The number of atoms in the critical nucleus as a function of the number of special OR's between the austenite nucleus and the surrounding ferrite grains.

Supplementary Table S1

Table S1: The values of the fitting parameter A and the variation in the S/C-ratio for the number of special OR's between austenite and ferrite.

OR	*A*	Variation in *S*/*C*-ratio
1	0.05±0.03	0.05 - 5.3
2	0.002±0.001	0.002 - 0.2
3	0.002±0.001	0.002 - 0.2
4	0.0004±0.00005	0.0004 – 0.0426

Intermediate results and discussion

Three intermediate results are used in fitting the classical nucleation theory to the measured nucleation rates: 1) the density of potential nucleation sites, 2) the austenite fraction, and 3) the driving force for nucleation.

Density of potential nucleation sites

The density of potential nucleation sites N_n as a function of temperature is estimated from the measurements in the following way. Before the ferrite-to-austenite transformation, the density of potential nucleation sites is equal to or higher than the number of nuclei that we experimentally observe at the end of the transformation. By increasing the temperature with one step of 2°C, the number of potential nucleation sites decreases by the number of actual nuclei that appear in that temperature interval during the experiment. An example of the density of potential nucleation sites $N_n^{'}$ that we measured in this way as a function of temperature is shown in Fig. S4 for the case that the austenite nuclei have 1 special OR with the neighboring ferrite grains. Here, we assume that the actual density of potential nucleation sites N_n changes with temperature in the same way as the measured density of potential nucleation sites $N_n^{'}$ (see supplementary Fig. S4), except for a constant scaling factor S, i.e. $N_n = S\,N_n^{'}$. A sigmoidal (Boltzmann) curve is fit to the measured $N_n^{'}$ (see supplementary Fig. S4). This sigmoidal curve for $N_n^{'}$ is used in Eqn. (3) to calculate $N_n^{'}$ at each temperature during the phase transformation.

Austenite fraction

In order to calculate the fraction transformed at each point during the phase transformation, all the diffraction images recorded during a Friedel 3D measurement were summed in order to

get powder-like diffraction images. After a radial integration step on the summed image, the formed austenite fraction (see supplementary Fig. S5) is calculated according to procedure described in ref. S1. A polynomial curve is fit to the measured austenite fraction. This polynomial curve for the austenite fraction is used in Eqn. (3) to calculate the austenite fraction at each temperature during the phase transformation.

Driving force for nucleation

The equilibrium temperature at which the ferrite to austenite transformation starts is calculated under ortho- and para-equilibrium conditions with the thermodynamic software package Thermo-Calc[28]. Ortho-equilibrium indicates that all alloying elements distribute according to equilibrium during the transformation. Para-equilibrium means that the carbon atoms have enough time to reach an equilibrium distribution, but the other atoms in the solid-solution do not have enough time to reach an equilibrium distribution. The transition temperature according to ortho-equilibrium conditions is 1118 K. The transition temperature according to para-equilibrium conditions is 1024 K. The experimentally observed transition temperature is 1105 K. From this we derive that the nucleation does not take place under ortho-equilibrium conditions, because the experimentally observed transition temperature is below the ortho-equilibrium transition temperature, which is physically not possible. The nucleation can take place under para-equilibrium conditions, because the experimentally observed transition temperature is above the para-equilibrium transition temperature.

The driving force for the transformation (under para-equilibrium conditions) as a function of temperature is calculated with the thermodynamic software package Thermo-Calc[28]. The result is shown in supplementary Fig. S6. Supplementary Fig. S6 shows that the driving force for the transformation is approximately $1.1 \cdot 10^7$ J/m^3 at 1105 K. A strain energy of 10^7 J/m^3 as

estimated in reference (*19*) could therefore explain the increase in the experimentally observed transition temperatures compared to the equilibrium para-equilibrium transition temperature.

Additional results and discussion

Physical interpretation values fit-parameter *A* of austenite nucleation rate

The experimentally observed nucleation rate that is shown in Fig. 4 is calculated by dividing the measured number of austenite grains at a particular temperature by the volume of the ‘cylinder’ and by the isothermal holding time of 10 minutes. However, the nucleation process ceased within shorter times than ten minutes. Therefore, the absolute nucleation rates that are reported in Fig. 4 are in fact higher by an approximately constant factor *C*. The relative change in nucleation rate with temperature remains approximately the same as reported in Fig. 4. The fitted *A*-parameter is therefore equal to $A = (S/C)Z\exp(-\tau/t)$, with $C \geq 1$ and $S \geq 1$.

The fitted values for the *A*-parameter are given in supplementary Table S1. In order to assess the physical meaning of these fitted values, we perform the following analysis. The value for the frequency factor is estimated to be $\beta^* = (kT/h)\exp[-Q_D/(kT)] \approx 229$ s^{-1} (*13*), for $T = 1115$ K and $Q_D = 3.93 \cdot 10^{-19}$ J (*26*). The incubation time is given by $\tau = \kappa/(2\pi\beta^* Z^2)$. Given that $0.5 < \kappa < 5$ (*4*) and $0.01 < Z < 1$ (*14*), the incubation time is $(7 \pm 15) \cdot 10^{-3}$ s. Therefore, the factor $\exp(-\tau/t) = 1.00000 \pm 0.00003$ for $t = 600$ s. From $\exp(-\tau/t) = 1$ and the fitting parameter *A*, we derive the variation in the *S*/*C*-ratio as given in supplementary Table S1. Generally, the *S*/*C*-ratio is below 1, except for OR1, where the *S*/*C*-ratio varies between 0.05 and 5.3. An *S*/*C*-ratio >1 means that $S > C$ and $S > 1$, which means that the density of potential nucleation sites is higher than the density of nuclei with OR1 that actually appeared in the experiment. In the case of OR1, *S* can be larger than 1, because there are a large number of potential nucleation sites in

the microstructure where austenite nuclei with OR1 can form, since there are essentially no crystallographic restrictions. However, there is an upper bound to *S*, because $C \geq 1$. The maximum for *S* is 5.3, which is obtained for the case $C = 1$. This corresponds to a maximum time t_{end} of 10 minutes at which nucleation ended during the isothermal holding.

Supplementary Table S1 shows a trend in which the *S*/*C*-ratio decreases with increasing number of OR's. This could be related to the fact that with increasing number of OR's the number of potential nucleation sites becomes more restricted due to increasing crystallographic restrictions. This means that the value for *S* decreases with increasing the number of OR's. The fact that the *S*/*C*-ratio is below 1 for OR's 2-4, means that $C > S$. In case the *S*/*C*-ratio = 0.2, which is the upper bound for OR2 and OR3, and $S = 1$, then $C = 5$, which means the nucleation process ended latest after a time t_{end} of 2 minutes. In case the *S*/*C*-ratio = 0.04, which is the upper bound for OR4, and $S = 1$, then $C = 23$, which means the nucleation process ended latest after $t_{\text{end}} = 26$ seconds.

Supplementary Fig. S7 shows the maximum time t_{end} at which the nucleation ended during isothermal holding as a function of the number of crystallographic OR's. Supplementary Fig. S7 shows a trend in which t_{end} decreases with increasing number of special OR's. This is logically related to Fig. 4, which shows that the activation energy for nucleation decreases with increasing number of special OR's. In other words, a lower activation energy for nucleation leads to a shorter time t_{end} in which the nucleation process ends during isothermal holding.

Number of atoms in the critical nucleus

Supplementary Fig. S8 shows the number of atoms n^* in the critical nucleus, which is given by[14] $n^* = 2\Psi/[\nu_\gamma(\Delta g_V - g_s)^3]$, where $\nu_\gamma = (1/4)(a_\gamma)^3$ is the atomic volume of iron in the austenite phase and a_γ = 0.366396 nm is the lattice parameter of the austenite. The values for Ψ are taken from Fig. 4. The driving force for nucleation is taken at the temperature at which the nucleation rate is maximum. Supplementary Fig. S8 shows that the number of iron atoms in the critical nucleus is about seven thousand in case $\Psi = 10^{-7}$ J^3/m^6 and $g_S = 10^7$ J/m^3. Given the uncertainty in the absolute value of Ψ, which varies between 10^{-4} to 10^{-8} J^3/m^6 as we change g_S from 0 to $1.1 \cdot 10^7$ J/m^3, respectively, the number of atoms in the critical nucleus lies between 2,500 and 10,000 atoms.

Details 3DXRD data-analysis

Raw Data: peak searching and fitting

A low threshold of 150 counts was used in order to identify regions consisting of diffracted intensity. After peak searching and fitting, almost 99 % of the diffraction spots were consistently identified. The global parameters of the experiment were determined from the diffraction data itself and no calibration sample was required, as described in references (24,25).

Indexing: correlating diffraction spots to individual grains

In order to keep the processing time low, indexing and refinement routines were executed on each Friedel 3D measurement with the following parameters: Diffraction spots belonging to the {200}-planes were chosen as starting points for indexing. Since the {110}-ring of α and {111}-ring of γ overlap, the diffraction spots belonging to these two rings were omitted from analysis in case both phases are present in the microstructure. In the analysis, a 5 μm step in the position in the sample and 0.2° step in the rotation of the diffracting plane normal were used. The margins used for the position on the detector and in ω were 60 μm and 0.4°, respectively. Detection of both the mixed and the un-mixed Friedel pairs is taken into account. All the grains for which the mean difference in ω was less than 0.06° and the mean difference in the position of the observed and the simulated diffraction spots on the detector was less than 25 μm were identified and stored as grains.

Identifying the grains in the 'cylinder'

Due to the horizontal size of the X-ray beam being smaller than the diameter of the sample during rotation, the part of the sample illuminated by the X-ray beam changed continuously, even though a smaller cylinder of diameter equal to the width of the beam was always in the

beam. Due to this, firstly, the set of grains at the edges of the smaller cylinder was different each time the grains diffract in different orientations of the sample. Secondly, for the grains located outside this cylinder, not all diffraction spots are observed because the grains are not in the beam during the entire angular scanning. Thus, three filtering steps were used here: (i) the minimum ratio of observed number of diffraction spots divided by the expected number of diffraction spots was chosen to be 0.8; (ii) the difference between grain sizes as calculated from a starting diffraction spot and the diffraction spots being matched during indexing was chosen to be less than 10 μm in grain radius and; (iii) a grain was accepted only if the refined centre-of-mass position of the grain was within the smaller cylinder defined by the beam size. In the case of the grains close to the edges of the cylinder, the uncertainty in the centre-of-mass position, the orientation and the volume of the grains is accordingly higher than for that for the grains located completely in the cylinder.

Detection limits

At each point during the phase transformation the grains were observed after the nucleation stage, because of the detection limit and because the Friedel 3D measurements were carried out only after the equilibrium phase fractions had been reached. The volume of the smallest grains that could be detected at any time during the experiment (determined by the threshold used) is ~ 120 μm^3 (~ 3 μm radius), so that all the grains above this size could successfully be identified. Thus, even though all the grains larger than 3 μm in radius could be identified, no nuclei of such small size were found because the nuclei had already grown before the Friedel 3D measurements were carried out. In the results shown here, the minimum size of the γ grains when first detected (calculated from volume assuming spherical shape) is between 4.8 to 12 μm, since some nuclei had already grown before the start of the Friedel 3D measurements. Compared to the much coarser initial α microstructure (average grain size of ~

105 μm) and the final γ microstructure (average grain size of ~ 98 μm), this is not expected to have a significant effect on identifying which are the neighboring ferrite grains close the moment an austenite grain nucleated. Moreover, we can determine at which temperature interval a nucleus forms, since the heat-treatment consists of discrete temperature steps ΔT and we anneal at a particular temperature until the moment that the austenite grains stop growing.

Identification of neighboring grains

In the experimental setup, diffraction images were recorded using the medium resolution FReLoN-4M detector, due to which the geometry of the grain boundaries could not be determined. However, it is not needed for this study to reconstruct the morphology of the grain boundaries, which in this case reduces the measurement time from many hours (if not days) to 13 minutes. The shape of the grains were approximated by ellipsoids with small aspect ratios. The strategy to identify which are the neighboring ferrite grains close the moment an austenite grain nucleated is as follows: The distance between the centre-of-mass of an austenite grain (at the time when it was first detected and thus relatively small) and that of each ferrite grain in the illuminated volume is calculated first including a factor to account for the uncertainty in the position of the grains. This distance is normalized by the radius of the corresponding ferrite grain to calculate a so-called *'sphere of influence'* of the ferrite grain. The ferrite grains for which this *sphere of influence* had a value smaller than 1.5 were identified as possible neighbours of the austenite grain under consideration, henceforth referred to as the candidate ferrite grains. This step makes sure that if the ferrite grain is identified as a neighbour in the end, the shape of the grain is still close to an ellipsoid with a maximum aspect ratio of 1.5. Repeated calculations showed that the choice of the maximum aspect ratio had no significant influence up to a value of 2.

For each of the candidate ferrite grains, the 3D space was divided into half. This was done by transforming the coordinates of the ferrite grains in such a way that the *z*-axis of position coordinates was aligned in the direction from the centre-of-mass of the austenite grain towards the candidate ferrite grain. In case the *sphere of influence* of the ferrite grain in question was less than the *sphere of influence* of the remaining ferrite grains (with positive value of transformed *z*-position) that are closer to the austenite grain (in absolute distance) as compared to the ferrite grain in consideration, the ferrite grain was identified as a neighbour of the austenite grain. In case there were ferrite grains with the *sphere of influence* smaller than the original ferrite grain, the absolute distance between the ferrite grains and the austenite grain was compared and the ferrite grain positioned at the smallest absolute distance from the austenite grain was saved as a neighbour of the austenite grain.

Reference

S1. N. H. van Dijk, A. M. Butt, L. Zhao, J. Sietsma, S. E. Offerman, J. P. Wright, and S. van der Zwaag, Thermal stability of retained austenite in TRIP steels studied by synchrotron x-ray diffraction during cooling, *Acta Mater.* **53**, 5439-5447 (2005).
